# A New Surgical Operation

**Published:** 1888-08-18

**Authors:** 


* "Fancy-Fair Religion; or, The World Converting Itself." By the Rev. J. Priestly Foster, M.A., Vicar of Oxenhall, Gloucester.

### A New Surgical Operation.

Among the matters of interest which were brought before the British Medical Association, at the recent Glasgow meeting, was an account by Mr. Brudenell Carter of a method which he had devised for opening the sheath of the optic nerve behind the eye, for the relief of pressure within this sheath and within the cavity of the skull. The brain is invested by firm membranes, which secrete a certain amount of fluid, and are continued down to the eye in the form of a sheath which surrounds the optic nerve; and, whenever the pressure within the cavity of the skull is increased, as by the growth of a brain tumour, or even by excess of secretion from the membranes themselves, a superabundance of fluid is apt to find its way down the nerve sheath to the level of the eye, to subject the optic nerve to injurious pressure, and, in many cases, to destroy the sight. It not infrequently happens that the pressure within the brain cavity may be increased by temporary or curable causes, which, nevertheless, continue in action sufficiently long to produce permanent blindness, even although the patient may, in other respects, recover. In view of these conditions it was suggested by Dr. de Wecker, of Paris, 16 or 17 years ago, that it might be possible to open the optic nerve sheath, and thus not only to relieve the nerve from pressure and to preserve it from injury, but also, on account of the position of the eye relatively to the brain cavity, to drain the latter by gravitation, and to relieve the brain as well as the eye. Dr. de Wecker made two endeavours to accomplish this object but he tried to feel his way to the optic nerve without the aid of sight, and to incise the sheath by means of an instrument carrying a concealed knife, capable of being projected by means of a spring. The risks of failure, and, still more, the risks of inflicting irreparable injury upon the nerve, were such that he only attempted his operation in two wellnigh hopeless cases, and only one attempt to follow his example has been recorded. Mr. Carter's attention was called to the matter last year by a case in which the diminution of pressure within the optic nerve sheath was manifestly desirable; and he devised a method of operating by which the sheath could be exposed to view, and the object attained with certainty, under the guidance of sight at every step of the process. He read before the Medical Society of London, last year, an account of the first case in which he operated, which was successful; and he read an account of three more cases at Glasgow, in one of which the result was negative, as far as sight was concerned; while in the other two the patients were not only quickly restored to useful vision, in one instance from complete, in the other from nearly complete blindness, but were at the same time relieved or cured of other symptoms, such as headache and sickness, arising from direct pressure on the brain. In his paper at Glasgow, Mr. Carter claimed for the new operation that it could be performed with certainty, and without risk either to life or to any important structure, and that it afforded a reasonable prospect of the preservation of sight in many forms of disease in which it is now habitually or frequently lost. As in the case of every new operation, time and further experience of its effects are required, in order to determine the precise limits of its usefulness. In the discussion which followed the paper, Mr. Bickerton, of Liverpool, said that, in consequence of reading the account of Mr. Carter's first case, he had himself performed the operation in two instances, in one of which temporary restoration of sight was followed by relapse, while in the second the ultimate issue was favourable.

